# Expression of the *ydaJKLMN* operon increases the adhesion of *Bacillus subtilis* spores to biotic and abiotic surfaces

**DOI:** 10.1128/aem.00086-26

**Published:** 2026-05-27

**Authors:** Marina De Stefano, Anella Saggese, Ezio Ricca, Loredana Baccigalupi

**Affiliations:** 1 Department of Biology, Federico II University, Naples, Italy; 2 Department of Molecular Medicine and Medical Biotechnology, Federico II University 208969, Naples, Italy; Washington University in St. Louis, St. Louis, Missouri, USA

**Keywords:** adsorption, spore structure, adhesion properties, exopolysaccharides

## Abstract

The adhesion of microbial cells to biotic and abiotic surfaces is generally mediated by molecules present on the cell envelope. In the case of spore forming bacteria, the exosporium, pilus-like appendages and surface-exposed molecules have been identified as mediators of the adhesion of spores of various species to different surfaces. Here, it is reported that in *Bacillus subtilis,* the expression of the *ydaJKLMN* operon increases the adhesion of spores to both biotic and abiotic surfaces. The *ydaJKLMN* operon codes for enzymes putatively involved in the production of an exopolysaccharide and is expressed in vegetative cells under stress conditions by the action of the alternative sigma factor of the RNA polymerase SigB. Although *ydaJKLMN* is not expressed in sporulating cells, its expression in vegetative cells increases the adhesion efficiency of spores. A model is presented suggesting that the exopolysaccharide synthesized by the YdaJKLMN enzymes, once secreted by vegetative cells, binds spores released by sporulating cells present in the same culture increasing their adhesion to biotic and abiotic surfaces. The *in vitro* observation that in a heterogeneous population, such as a *B. subtilis* culture, a subpopulation of cells secretes molecules that modify the phenotype of another set of cells could reflect a common behavior of microbial populations in nature.

**IMPORTANCE:**

For their ability to efficiently and tightly bind industrial and medical equipment, bacterial spores are a serious concern for food and pharmaceutical industries as well as for health care institutions. Spore adhesion to surfaces is due to outermost molecules or, in the *Bacillus cereus* group species, to pilus-like appendages. This study reports that in a heterogenous culture of *Bacillus subtilis,* molecules secreted by vegetative cells, not committed to sporulation, increase the efficiency of adhesion of spores produced by sporulating neighbor cells. Such *in vitro* observation could reflect a common behavior of microbial populations in a natural environment, with the products of the metabolic activities of some cells used by other cells to expand their own biological functions.

## INTRODUCTION

Bacterial (endo)spores of the *Bacillus* and *Clostridium* genera are quiescent and highly resistant cells that withstand conditions that would be lethal for other cell forms. Spores are ubiquitous in nature, being found in almost every environment, including soil, air, water, plant material, and the intestinal tract of insects and animals (1, 2). Such a wide distribution is mainly due to the spore resistance properties that allow their survival at extreme conditions of temperature and/or pH, to the presence of UV radiation, toxic chemicals, and lytic enzymes (1). In addition to the resistance properties, also the ability of spores to adhere to biotic and abiotic surfaces contributes to their environmental distribution and success (1–3).

For their resistance to standard sanitizing and cleaning procedures and their ability to efficiently and tightly bind industrial and medical equipment, spores are a serious concern for food and pharmaceutical industries as well as for health care institutions (4, 5). Similarly, health concerns come from spores of pathogenic species, including *B. anthracis, B. cereus, C. botulinum,* or *C. difficile*, able to bind animal cells and tissues (6, 7). On the other hand, for spores of commensal or probiotic species that inhabit the intestinal tract of animals exerting beneficial health roles (2, 8), their ability to survive gastric conditions and adhere to intestinal cells and mucosal surfaces are key aspects of the spore-host beneficial interaction (9). Adhesion to mucin and intestinal epithelial cells is, indeed, essential to avoid a fast transit through the gastro-intestinal tract, thus favoring the germination of the spores in the intestine, the colonization of that niche, and the beneficial health effects (8). The adhesion properties of the spore are also relevant for their ability to bind heterologous molecules when these are highly concentrated in the extracellular environment (10). Such biotechnologically relevant properties have also been exploited in the development of spores of non-pathogenic organism as a delivery system for antigens and enzymes (11).

Spore adhesion to surfaces has been studied in various species, and it has been shown that different species have different surface structures and different adhesion properties (3, 12, 13), thus allowing the hypothesis that such properties are an adaptation evolved to facilitate productive interactions between spores and their specific environments (12). This hypothesis is also supported by the observation that the spore surface structure and, as a consequence, the adhesion properties of the spore are influenced by the conditions of cell growth and spore formation (14, 15). In some spore formers, such as *B. megaterium, B. cereus, B. anthracis*, *C. difficile,* and *C. botulinum,* the exosporium is the spore outermost layer and it has been recognized as responsible of the spore adhesion (16–18). In the *B. cereus* group species, in addition to the exosporium, the adhesion of spores to surfaces is also mediated by pilus-like appendages (19–21). In *B. subtilis*, the model system for spore formers, the exosporium is not present and the outermost layer is the crust, a structure formed by proteins and glycans (22, 23). These glycans, containing rhamnose, galactose, quinovose, glucosamine, muramic lactam, and legionaminic acid residues (24), modulate the relative hydrophilicity (25, 26) and the adhesion properties of the spore (27).

This study analyzes the adhesion properties of spores of four *B. subtilis* strains, two type strains, 168 and NCIB 3610, the prototrophic laboratory collection strain PY79 and an intestinal isolate with probiotic potential, SF106 (28, 29). SF106 spores have been previously reported to have high adhesion properties, being able to bind mucin and heterologous molecules (30) more efficiently than other strains of the same species. PY79 spores were significantly less adhesive than spores of the other three strains which all behaved similarly. PY79 is a 168-derived strain, known to differ from 168 in at least four large deletions that are believed to have occurred as part of the domestication process of the lab strain (31). A comparative genomic analysis indicated one of such large deletions of 17 kb as the only chromosomal region lacking in PY79 but present in all three other strains here analyzed. Bioinformatic analysis of the 17 kb region, together with the analysis of the adhesion of spores of mutant strains, indicated that the presence of the *ydaJKLMN* operon increases the efficiency of adhesion to biotic or abiotic surfaces. This five-gene operon codes for a putative glycosyl hydrolase (*ydaJ*)*,* a c-di-GMP receptor (*ydaK*), and three genes (*ydaLMN*) coding for enzymes involved in the production/secretion of an exopolysaccharide (EPS) involved in cell aggregation and biofilm formation (32).

## RESULTS

### Spores of *B. subtilis* strains lacking a 17 kb chromosomal region have low adhesion properties

**Fig 1:**
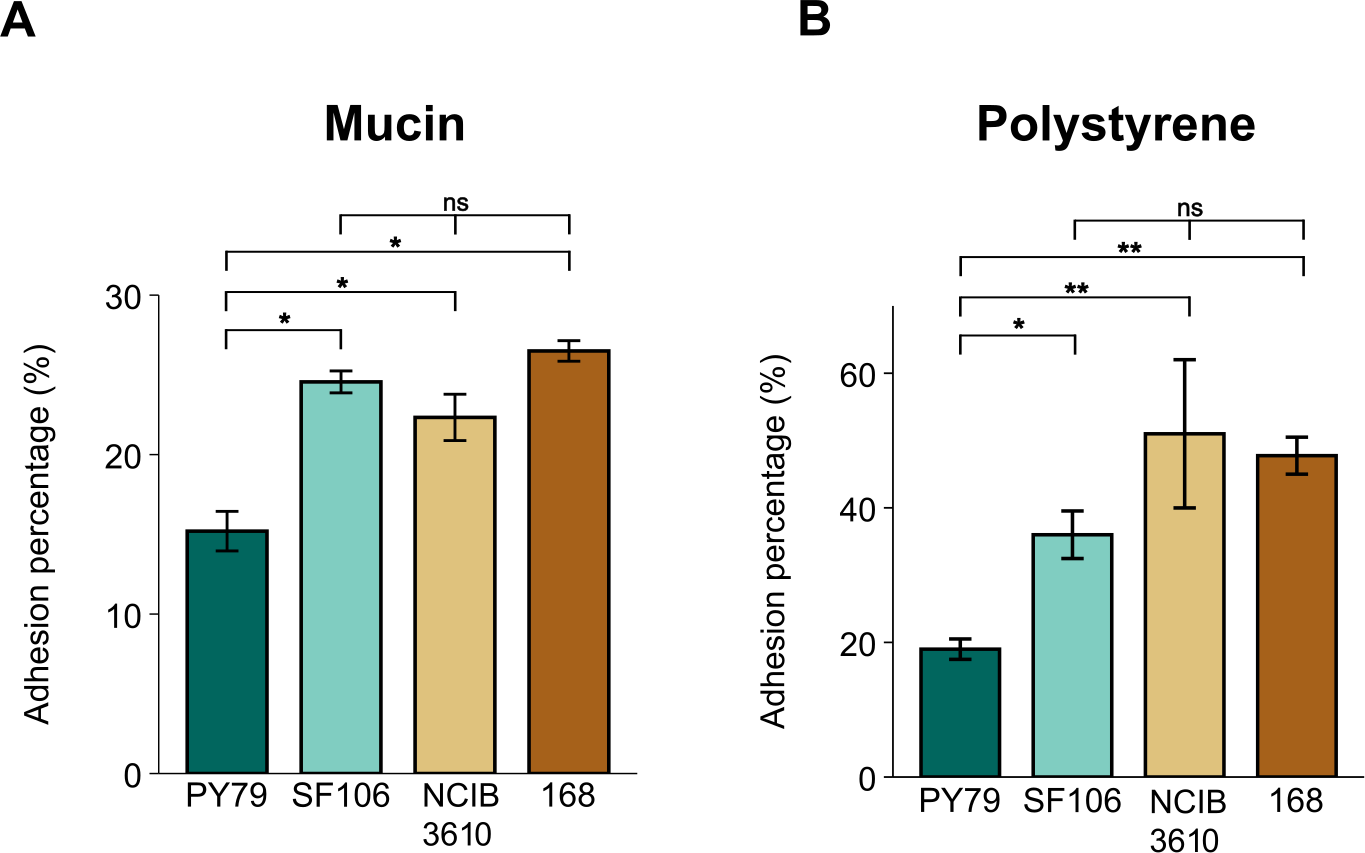
Adhesion of spores of different strains of *B. subtilis* (PY79, SF106, NCIB 3610 and 168) to mucin (**A**) and to polystyrene (**B**). The data represent the mean of three independent experiments, and the error bars are the standard error of the mean (ns: differences not statistically significant; *P < 0.05, **P < 0.005).

The adhesion properties of spores of four strains of *B. subtilis* were analyzed and compared using mucin and polystyrene as models of a biotic and an abiotic surface, respectively. As shown in Fig. 1, spores of the prototrophic lab strain PY79 (33) showed a significantly lower adhesion to both mucin (panel A) and polystyrene (panel B) than spores of two *B. subtilis* type strains, NCIB 3610 and 168, and of the intestinal isolate SF106 (34).

**Fig 2:**
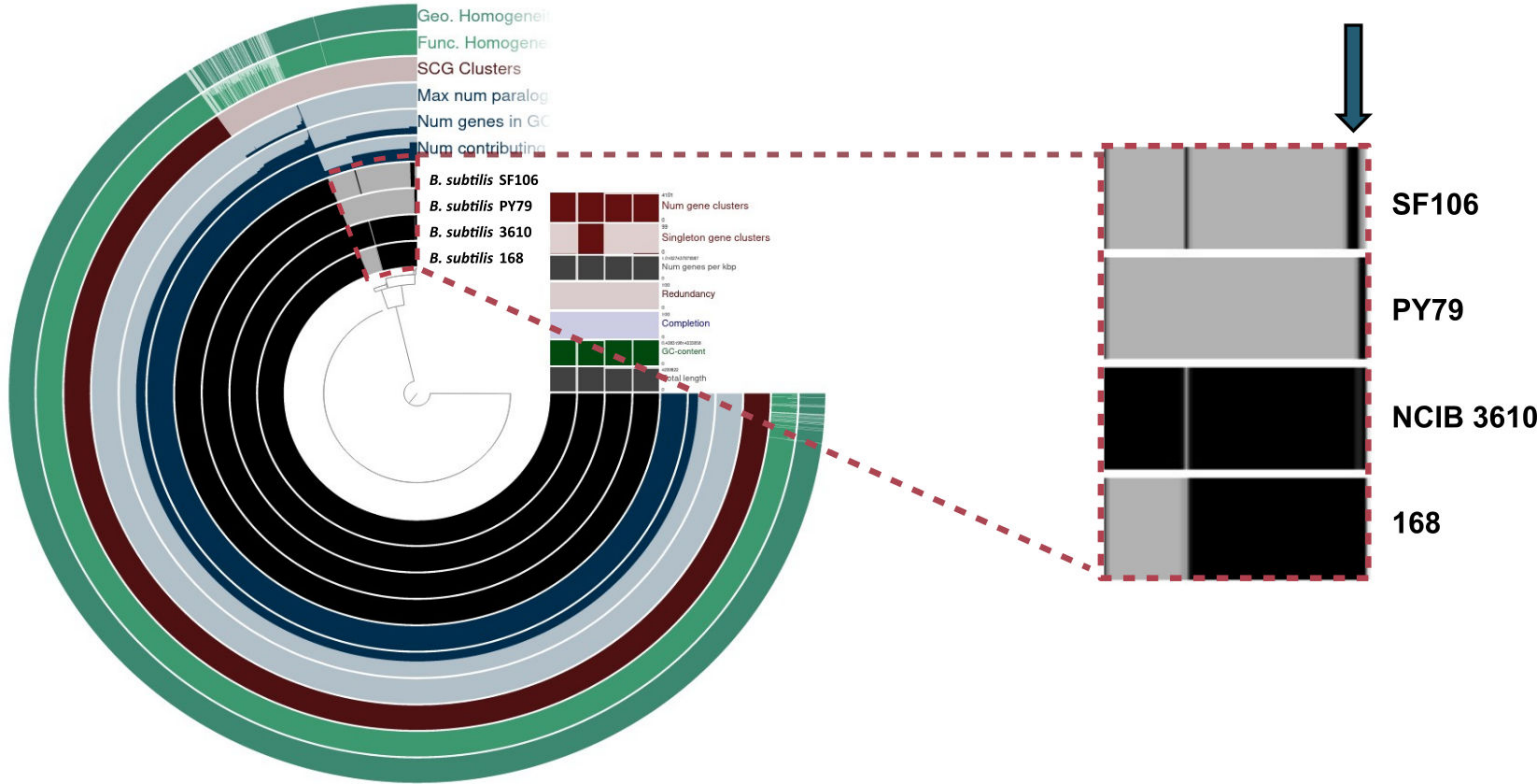
Genomic analysis of strains NCIB 3610, 168, PY79, and SF106. Anvi’o representation of the pangenome of the four *B. subtilis* strains generated with the items order in the presence/absence (D: Euclidean; L: Ward). Each black layer represents the genome of a strain. Boxed are the differences observed between the genomes of the four strains. In black and gray regions, respectively, present or absent in the indicated strain. The blue arrow indicates the only chromosomal region present in NCIB 3610, 168, and SF106 and absent in PY79.

**Fig 3:**
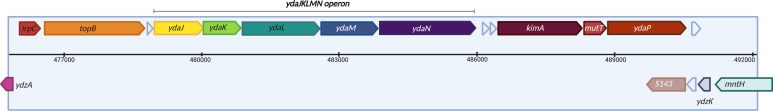
Genes carried in the 17 kb chromosomal region present in NCIB 3610, 168, and SF106 and lacking in PY79. Numbers refer to positions on the *B. subtilis* chromosomal map (35). [Created in BioRender. De Stefano, M. (2026) https://BioRender.com/05noiqk]

A comparative genomic analysis of the four strains was performed to evaluate whether genomic differences correlated with the different adhesion properties. As shown in Fig. 2, the genomes of the four strains were very similar with only minor differences. However, one chromosomal region was present in the genomes of the adhesive strains NCIB 3610, 168, and SF106 but absent in the weakly adhesive strain PY79 (blue arrow in Fig. 2). This chromosomal region is approximately 17 kb and has been previously described as lacking in strain PY79 but present in strains 168 and NCIB 1036 (31, 35). This 17 kb region is bordered by the *ydzA* and *mntH* genes, respectively, coding for a 10.6 kDa protein of unknown function and a manganese transporter (35), and contains 11 other genes (35) (Fig. 3). These are the *lrpC* and *topB* genes, respectively, coding for the DNA-binding protein LrpC and for the DNA topoisomerase III, the five-gene *ydaJKLMN* operon, coding for proteins involved in the synthesis of an extracellular polysaccharide, and the *kimA, mutA,* and *ydaP* genes*,* coding for a high affinity potassium transporter, an RNA pyrophosphohydrolase and a pyruvate:quinone oxidoreductase, respectively (Fig. 3) (35). On the opposite DNA strand is the *ydzK* gene that codes for a 9.6 kDa putative protein of unknown function (Fig. 3) (35).

### An insertional mutation in the *ydaJKLMN* operon affects spore adhesion to mucin and polystyrene

Based on the available information (35), of all proteins encoded by the 17 kb region, those encoded by the *ydaJKLMN* operon appear as the more likely related to the spore adhesion properties. To evaluate the role of the products of the *ydaJKLMN* operon in the adhesion of spores to mucin or polystyrene, a null mutation inactivating the entire operon was constructed. A 698 bp DNA fragment totally internal to the *ydaJ* gene was PCR amplified (see Materials and Methods), cloned into the integrational vector pER19 (36) to create plasmid pSO16. This plasmid was used to transform competent cells of SF106 with selection for chloramphenicol-resistance (Cm^R^), conferred by the integrational plasmid. Cm^R^ transformants were expected to arise by integration of pSO16 by single-reciprocal (Campbell-type) recombination between the *B. subtilis* DNA insert in the plasmid and the corresponding region of the chromosome (Fig. S1). One of the clones, AZ765, was tested by PCR (see Materials and Methods) to verify the site of insertion of the *cm* gene (data not shown) and selected for further studies.

**Fig 4:**
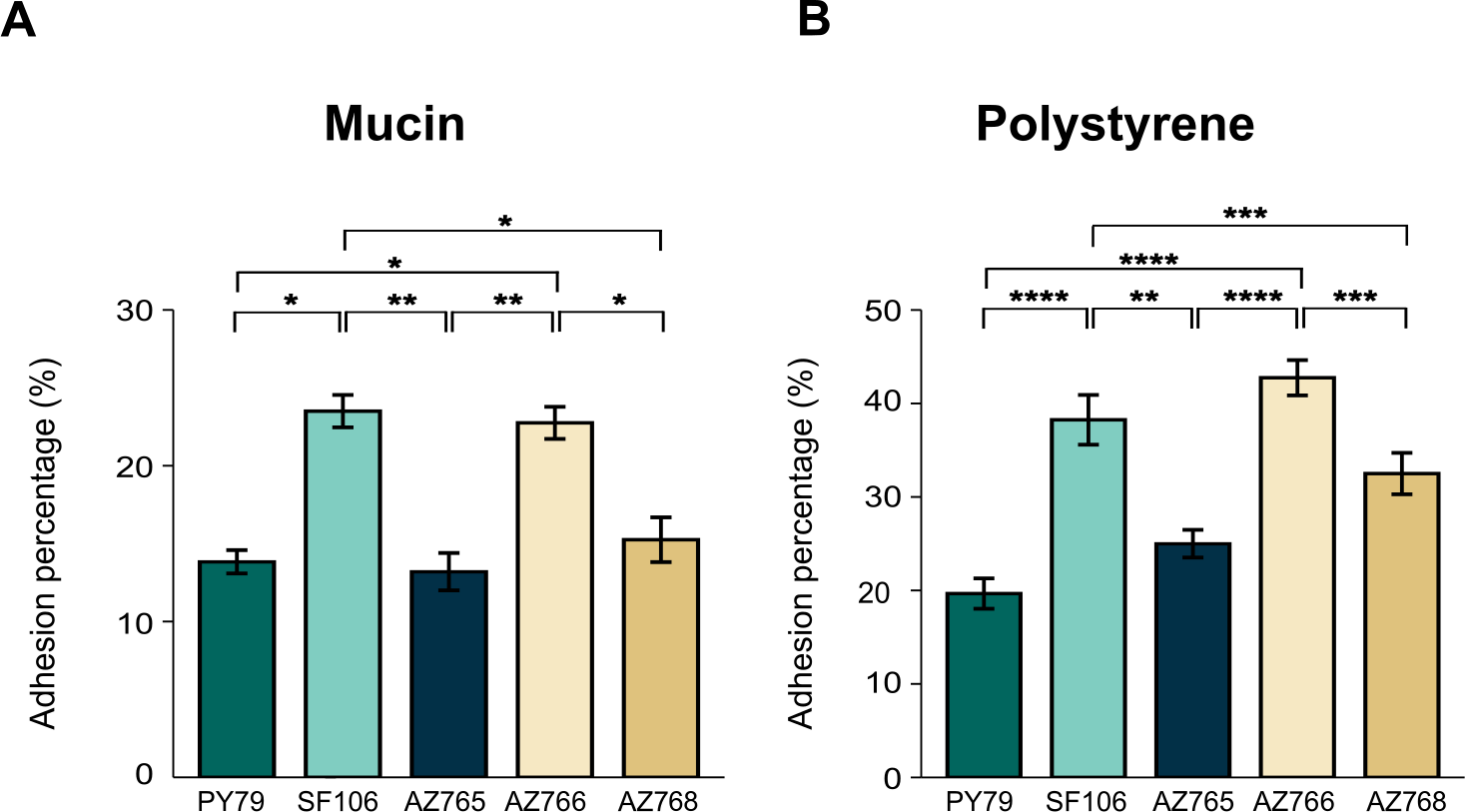
Adhesion of spores of different strains of *B. subtilis* to mucin (**A**) and to polystyrene (**B**). The data represent the mean of three independent experiments, and the error bars are the standard error of the mean (*P < 0.05; **P < 0.005; ***P < 0.001; ****P < 0.0001).

Purified spores of strain AZ765 showed an adhesion activity to mucin or polystyrene lower than spores of the isogenic parental strain (SF106) and similar to those of the PY79 strain (Fig. 4), suggesting that the products of the *ydaJKLMN* operon somehow mediated the adhesion of spores to both biotic or abiotic surfaces.

### The *ydaJKLMN* operon increases the adhesion of PY79 spores

The results of Fig. 4 also suggested that the low adhesion of PY79 spores could be due to the lack of the *ydaJKLMN* operon. To test this possibility, chromosomal DNA was extracted from strain BKK04260 (obtained from the *Bacillus* Genetic Stock Centre) and used to transform competent cells of strain PY79. BKK04260 is an 168 derivative carrying a kanamycin-resistance cassette integrated in the *topB* gene (*topB::kan*), included in the 17 kb region just upstream of the *ydaJKLMN* operon (Fig. 3). Kanamycin-resistant clones were expected to carry the selected marker and flanking chromosomal parts, necessarily extending over the borders of the 17 kb region lacking in PY79 to allow the transformation event. One of the clones, AZ766, was tested by PCR for the presence of the *ydaJKLMN* operon (see Materials and Methods) and selected for further studies. Competent cells of strain AZ766 (PY79 carrying the 17 kb region) were transformed with plasmid pSO16 with selection for Cm^R^ conferred by the plasmid as described above, to inactivate the *ydaJKLMN* operon, yielding strain AZ768 (PY79 carrying the 17 kb region with a null mutation in *ydaJ*). Purified spores of both AZ766 and AZ768 strains were analyzed for their mucin- or polystyrene-binding activities. As shown in Fig. 4, the presence of the *ydaJKLMN* operon in PY79 (strain AZ766) increased the adhesion efficiency of the spores to both biotic and abiotic surfaces and such increase was abolished by the inactivation of *ydaJKLMN* operon (strain AZ768).

### The products of *ydaJKLMN* expression modify the spore surface

The five products of the *ydaJKLMN* operon, responsible for the synthesis of an EPS involved in cell aggregation and biofilm formation (32), are present in most *B. subtilis* strains (not shown) and are also conserved in other *Bacillus* species (Table 1). While in some species, such as, for example, *B. licheniformis, B. safensis,* and *B. velenzensis,* all five YdaJ-N proteins are present and conserved, other species only have four of the five products (Table 1). To evaluate whether the *ydaJKLMN*-dependent increase in the efficiency of adhesion of spores to surfaces was actually due to an EPS present on the spore surface, a lectin-based approach was followed (37). A panel of four commercial lectins (see Materials and Methods) was reacted with the same number of purified spores of strains PY79, SF106, AZ765, AZ766, and AZ768. As shown in Table 2, lectin GSL-1 did not recognize polysaccharides present on PY79 spores, while it recognized sugars on spores of all strains carrying a functional copy of the *ydaJKLMN* operon. Since GSL-1 has an exclusive affinity for α-D-galactosyl (α-D-Gal) and α-N-acetyl-D-galactosaminyl (GalNAc) residues (37), results reported in Table 2 indicate that an EPS containing α-D-Gal and/or GalNAc residues is synthesized by the products of the *ydaJKLMN* operon and is assembled around mature spores.

**TABLE 1:** Amino acid identity (%) of the products of the *ydaJKLMN* operon in *Bacillus* species other than *B. subtilis*

Organism	Amino acid identity (%)				
	YdaJ	YdaK	YdaL	YdaM	YdaN
*B. licheniformis*	74.6	81.51	84.68	93.09	71.02
*B. sonorensis*	71.98	76.68	78.21	94.05	70.23
*B. velezensis*	65.59	99.08	79.62	96.14	99.45
*B. safensis*	46.28	64.1	43.32	81.66	43.69
*Aeribacillus kexueae*	42.39	34.98	36.89	76.68	35.02
*Lysinibacillus*	38.32	41.58	38.72	73.8	29.07
*Pueribacillus sp*.	41.54	33.77	39.86	73.67	30.83
*B. swezeyi*	72.8	81.98	75.92	–^*a*^	69.52
*B. atrophaeus*	70.6	77.74	76.8	–	68.56
*Virgibacillus*	*–*	94.83	41.42	73.58	31.3
*B. paralicheniformis*	*–*	76.33	82.62	92.13	70.62
*B. altitudinis*	45.04	67.39	48.09	–	41.19
*B. halotolerans*	39.51	31.2	87.35	–	28.12
*Heyndrickxia oleronia*	39.3	–	45.48	77.86	29.37
*B.massiliigorillae*	40.06	33.1	–	77.38	29.42
*Sporosarcina* sp.	40.18	–	40.81	75.24	29.38
*Pseudogracilibacillus*	35.69	36.67	–	73.32	29.82
*Peribacillus asahii*	38.74	32.34	34.6	71.67	–
*B. pumilus*	44.97	59.41	43.26	–	40.09

^*a*^ –, no similarity found.

**TABLE 2:** Lectin-binding analysis^*a*^

	PY79	SF106	AZ765	AZ766	AZ768
WGA	+	+	+	+	+
LCA	+	+	+	+	+
PSA	+	+	+	+	+
PHA-L	+	+	+	+	+
GSL-1	−	+	−	+	−

^*a*^ Wheat germ agglutinin (WGA): *N*-acetylglucosamine; *Lens culinaris* agglutinin (LCA): α-linked mannose residues; *Pisum sativum* agglutinin (PSA): α-linked mannose-containing oligosaccharides; *Phaseolus vulgaris* leucoagglutinin (PHA-L): tri- and tetra-antennary N-glycans with (1, 6)-branching; *Griffonia simplicifolia* lectin I (GSL-I): α-D-galactosyl and α-N-acetyl-D-galactosaminyl (GalNAc) residues. Presence (+) or absence (−) of fluorescence signals after 400 ms of exposure time.

### A null mutation in the *ydaJKLMN* operon affects the spore adsorption of heterologous molecules

**Fig 5:**
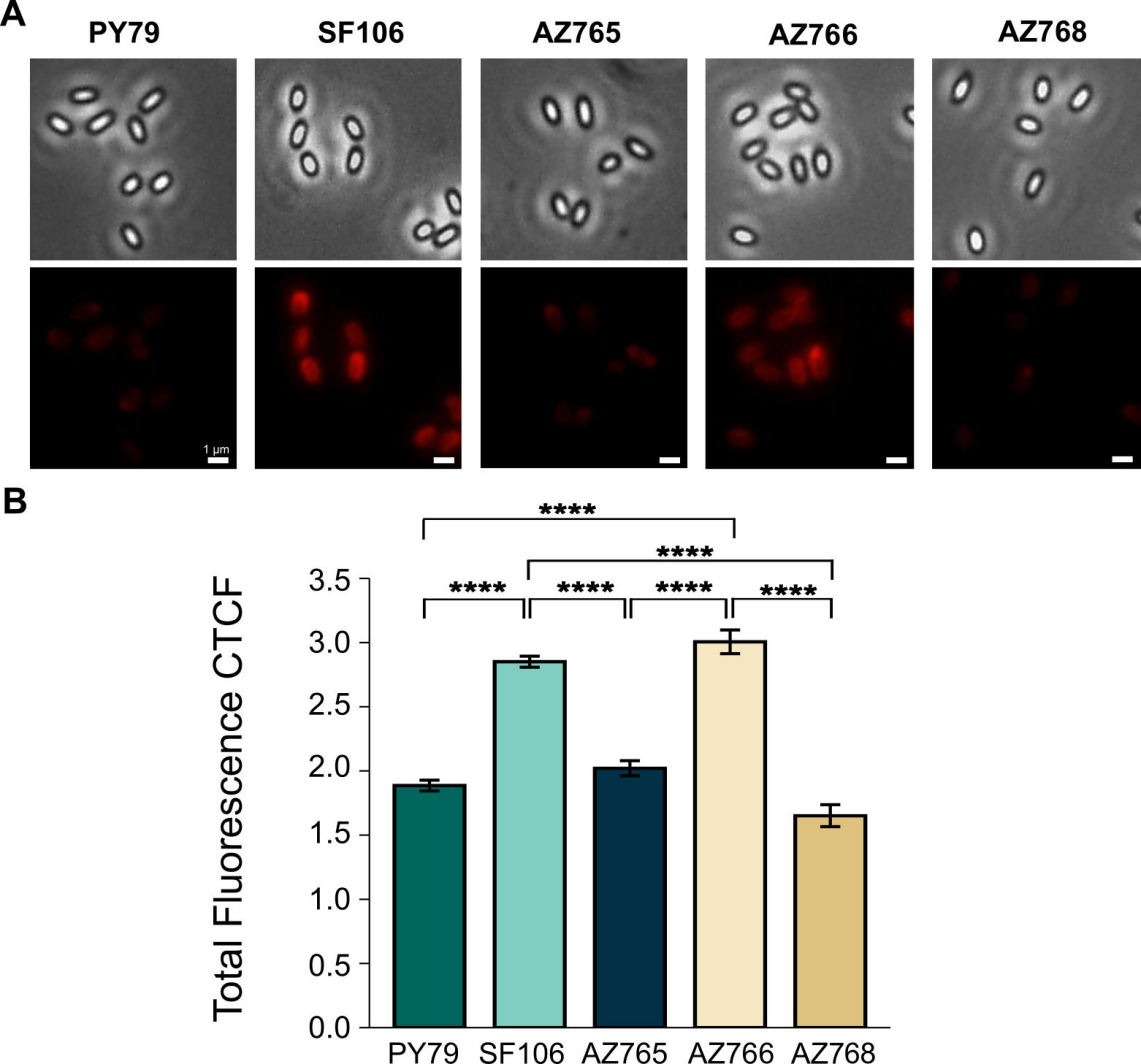
Efficiency of mRFP adsorption by spores of the indicated strains. (**A**) Microscopy analysis of spores adsorbed with 2 μg of mRFP in sodium citrate buffer at pH 3.5. Phase contrast and fluorescence images (respectively, upper and lower pictures for each strain) of spores of RFP adsorbed spores. Exposure time 500 ms, Scale bar 1 μm. (**B**) Quantitative analysis of fluorescence of 80 spores for each strain by ImageJ software (see Materials and Methods). The *X*-axis indicates the analyzed strains, the *Y*-axis shows the total corrected cellular fluorescence value. The data represent the mean of three independent experiments, and the error bars are the standard error of the mean (****P < 0.0001).

A previous study reported that SF106 spores adsorbed an enzyme, the β-galactosidase of *Alicyclobacillus acidocaldaricus,* and an antigen, the B subunit of the heat-labile toxin of *Escherichia coli,* more efficiently than PY79 spores (30). To test whether the adsorption of heterologous molecules was also influenced by the *ydaJKLMN* operon, spores of strain PY79, SF106, AZ765, AZ766 (PY79 *topB::kan*), and AZ768 (PY79 *topB::kan ydaK::cm*) were tested for the efficiency of protein adsorption by using the red fluorescent protein (RFP) (38) as a model. Purified spores were reacted with purified RFP and analyzed by fluorescence microscopy and flow cytometry, as previously described (38). SF106 spores adsorbed RFP more efficiently than PY79 and the inactivation of the *ydaJKLMN* operon of SF106 abolished this effect (Fig. 5). Similarly to what was observed with the adhesion to mucin or polystyrene, the acquisition of the *ydaJKLMN* operon was sufficient to increase the adsorption efficiency of PY79 to levels of SF106 (Fig. 5).

### Expression of the *ydaJKLMN* operon

Transcription of the *ydaJKLMN* operon is due to the alternative sigma factor of the RNA polymerase SigB (35, 39), which is known to control the expression of over 200 genes mainly involved in stress response but also in biofilm development and sporulation (40). To analyze the expression of the *ydaJKLMN* operon, DNA fragments of 698 bp and 742 bp internal to the promoter-proximal (*ydaJ*) and promoter-distal (*ydaN*) coding parts of the operon were independently cloned into plasmid pJM783 carrying the *lacZ* gene of *Escherichia coli,* yielding plasmid pMDS1 and pMDS3, respectively. These plasmids were then used independently to transform competent cells of SF106. Transformants were the result of a single reciprocal (Campbell-like) recombination between DNA present on the SF106 chromosome and on plasmid pMDS1 or pMDS3. The resulting strains AZ769 and AZ770, containing the transcriptional fusion *ydaJ::lacZ* or *ydaN::lacZ,* were used to evaluate *ydaJKLMN* expression by monitoring the β-galactosidase (β-Gal) activity due to the expression of the *lacZ* gene. Both gene fusions were also transferred by chromosomal-mediated transformation into strains AZ771 or AZ772, respectively, lacking SigB or Spo0A, yielding strains AZ773 (*sigB::neo ydaJ::lacZ*), AZ774 (*sigB::neo ydaN::lacZ*), AZ775 (*spo0A::neo ydaJ::lacZ*), AZ776 (*spo0A::neo ydaN::lacZ*) (Table S1).

**Fig 6:**
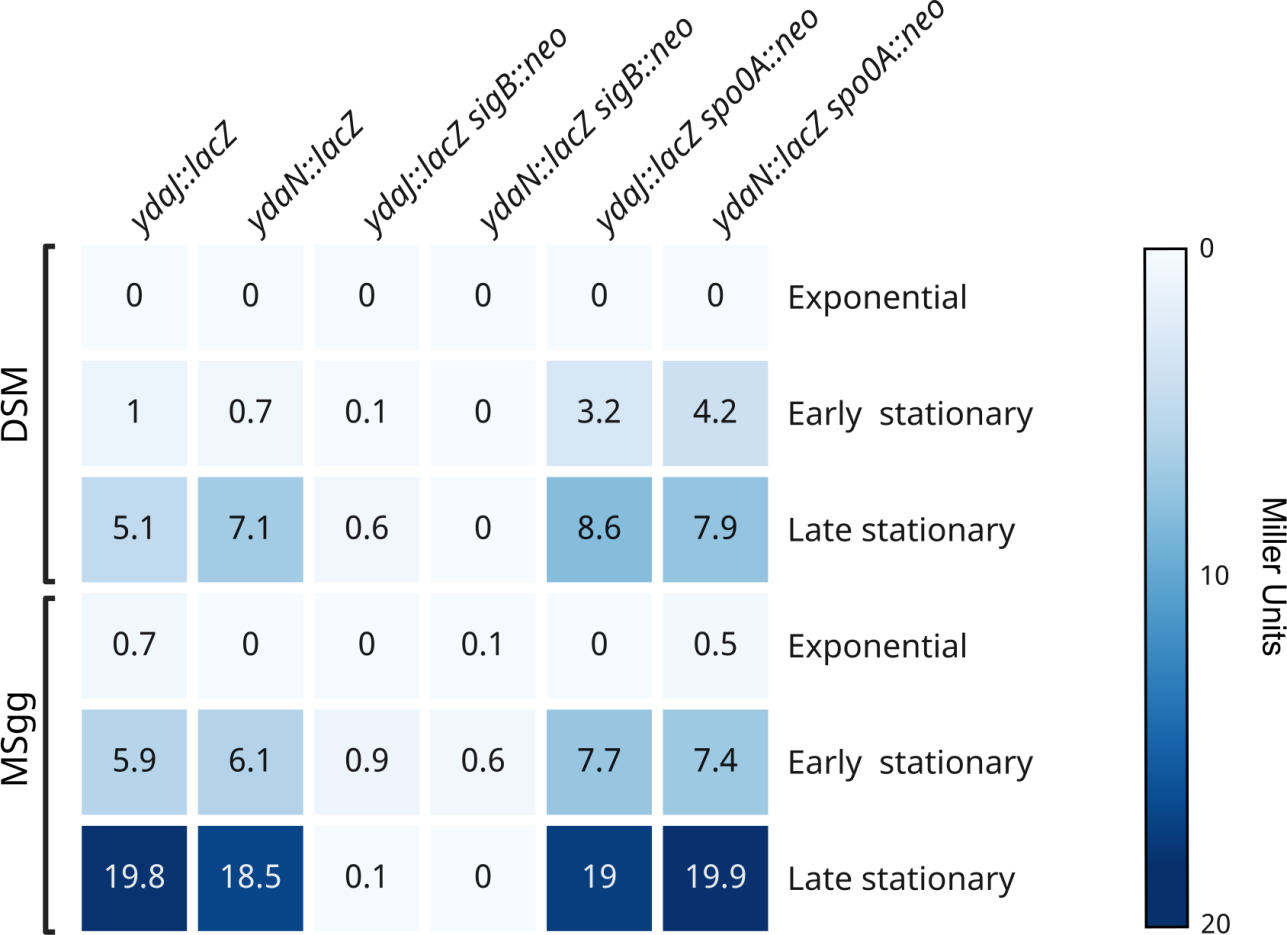
β-Galactosidase activity measured in strains carrying the *ydaJ::lacZ* or *ydaN::lacZ* gene fusion in an otherwise wild-type strain or in strains lacking SigB (*sigB::neo*) or Spo0A (*spo0A::neo*). Exponential, early- and late-stationary cells were collected after 4 h and then 20 and 40 h after the end of the exponential growth, respectively. Reported values represent the mean Miller Units from three independent biological experiments (each in triplicate). Statistical significance was assessed by comparing the slopes of linear regression models describing expression kinetics for each strain. As reported in Tables S3 and S4, the only statistically significant differences were in SigB-lacking strains when compared with wild-type or *spo0A*-lacking strains.

In a sporulation-inducing medium (DSM), both gene fusions showed a similar and extremely weak β-Gal activity, measurable only at a late stationary phase of growth (40 h after the end of the exponential growth) (Fig. 6). As expected, the β-Gal activity of both fusions was totally dependent on the presence of SigB while it was not dependent on the presence of an intact *spo0A* gene and, therefore, on the induction of sporulation (Fig. 6). In a biofilm-inducing medium (MSgg), the β-Gal l activity of both fusion strains was three- to fourfold higher than in DSM, was measurable during the early stationary phase of growth, and was further increased in a late stationary phase. Also in MSgg, the β-Gal activity of the two strains was similar, totally abolished in a SigB lacking strain and independent from Spo0A (Fig. 6).

### The SigB-dependent *ydaJKLMN* operon affects the adhesion of spores

**Fig 7:**
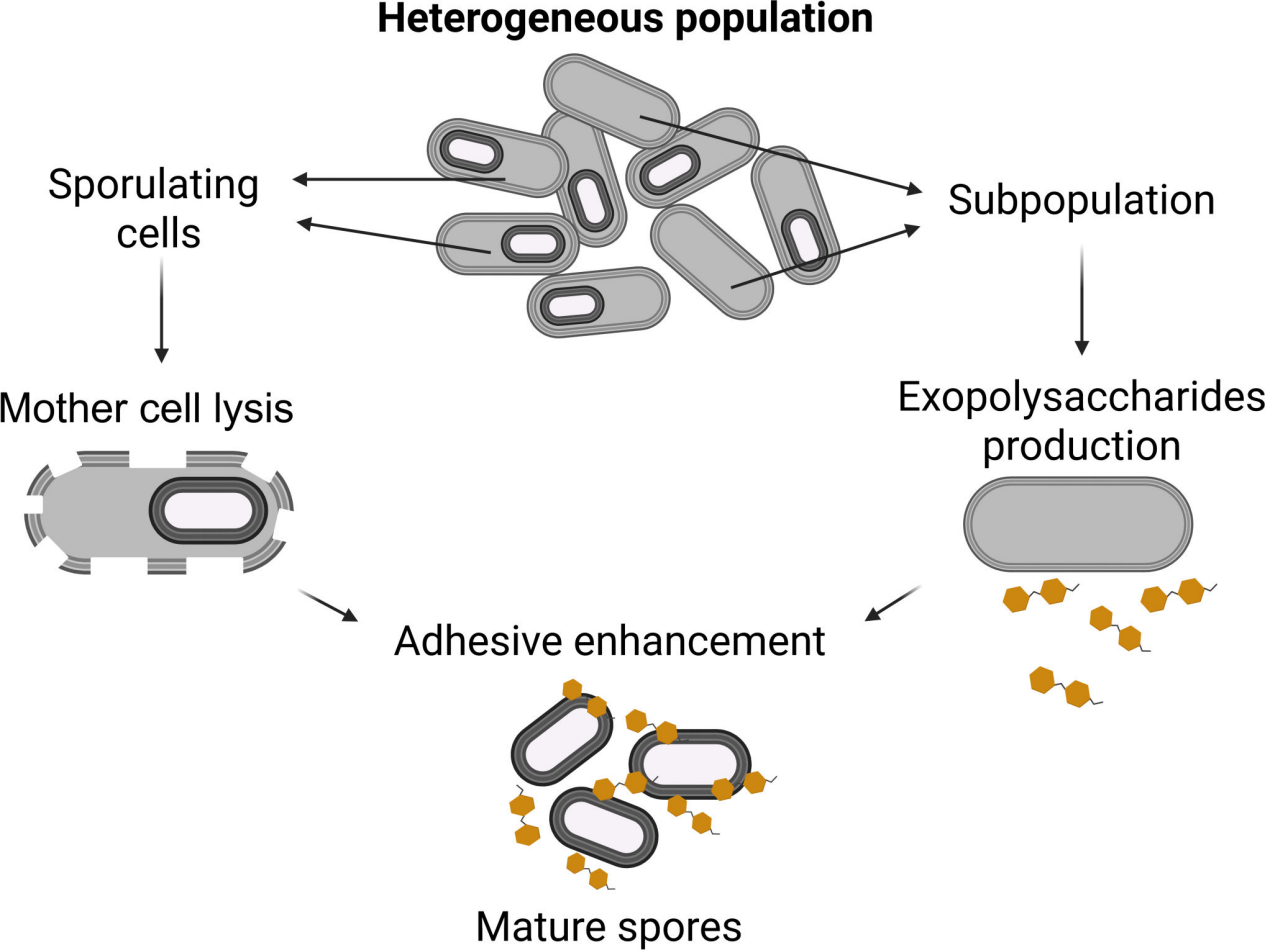
Working model. In a sporulation-inducing medium (DSM), a heterogeneous population is present (41, 42) and only cells not committed to sporulate express the *ydaJKLMN* operon, producing an EPS. The secreted EPS binds to spores released by sister cells committed to sporulate. [Created in BioRender. De Stefano, M. (2026) https://BioRender.com/chy3qit]

Taken together results so far reported indicate that the *ydaJKLMN* operon is responsible of the spore adhesion properties even though it is only weakly expressed in sporulation conditions under the control of the stress-specific sigma factor of the RNA polymerase SigB and with no involvement of sporulation-specific factors. A possible scenario is schematically illustrated in Fig. 7: during spore formation in DSM, a heterogeneous population of cells is present in the liquid culture, with the majority of cells committed to form spores and few cells that have not entered into sporulation. Only these cells would express the *ydaJKLMN* operon, synthesizing and releasing polysaccharides. These molecules would bind the surface of mature spores once these are released by the lysis of the mother cell at the end of the sporulation process, making their surface more adhesive to biotic and abiotic surfaces.

**Fig 8:**
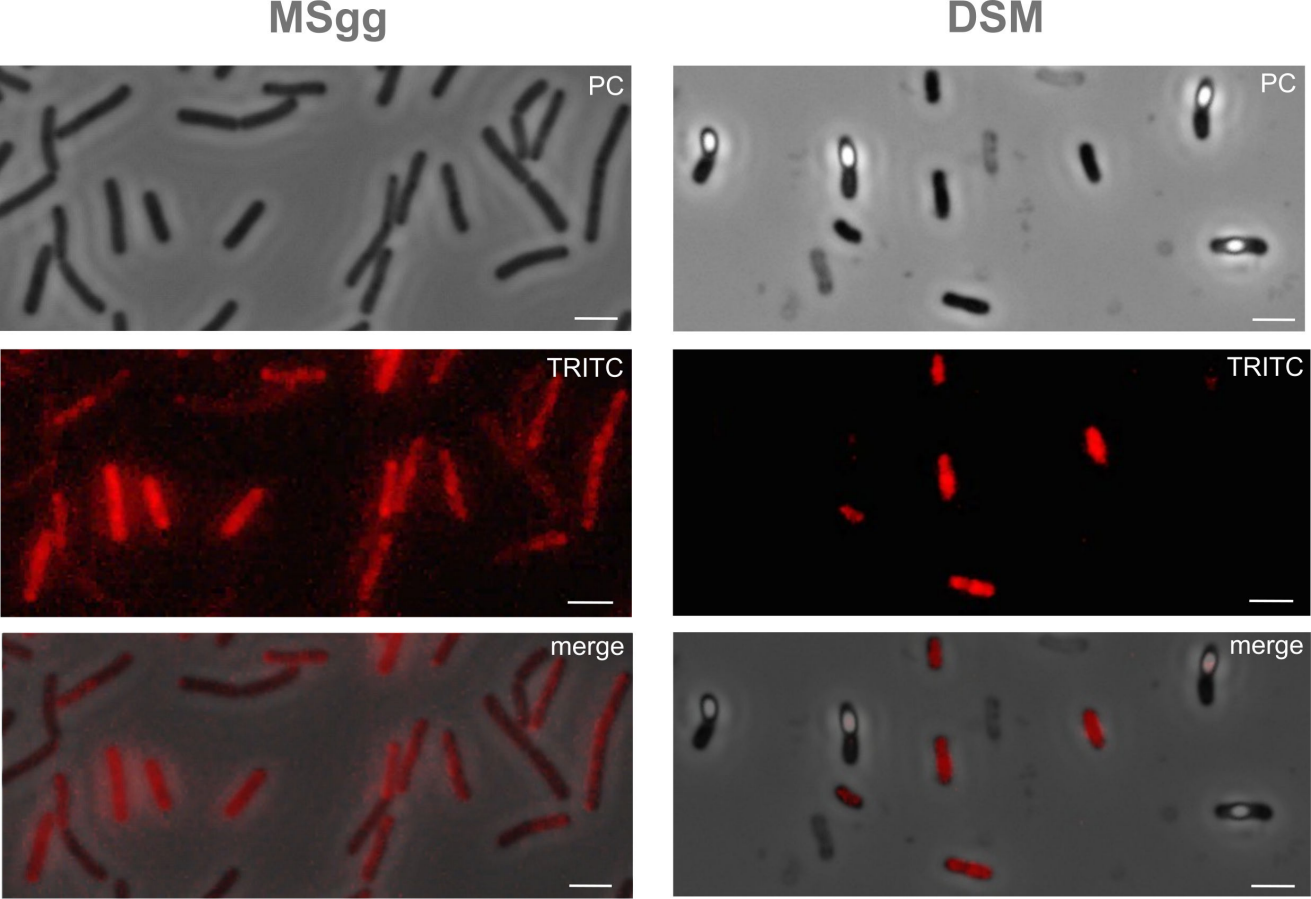
Fluorescence microscopy analysis of growing (left panels) or sporulating (right panels) cells of the SF106 strain carrying the *ydaJ::rfp* fusion. The same microscopy fields are reported using phase contrast (PC) and fluorescence (TRITC) microscopy, with exposure time of 800 ms (Size bar = 4 µm).

To test whether the *ydaJKLMN* operon is only expressed in cells not committed to sporulation, a single-cell approach was followed. A DNA fragment of 678 bp coding for the monomeric red fluorescent protein (mRFP) of the coral *Discosoma* sp. (43) was cloned into plasmid pSO16, carrying the ydaJ coding part, yielding plasmid pMDS10. This plasmid was then used to transform competent cells of SF106. Transformants were the result of a single reciprocal (Campbell-like) recombination between DNA present on the SF106 chromosome and on plasmid pMDS10. The resulting strain AZ804 containing the *ydaJ::rfp* fusion was used to visualize *ydaJKLMN-*expressing cells. As shown in Fig. 8, while in MSgg medium all cells were fluorescent, in sporulation conditions (DS medium), sporangia were not fluorescent and only cells not committed to sporulation appeared red.

**Fig 9:**
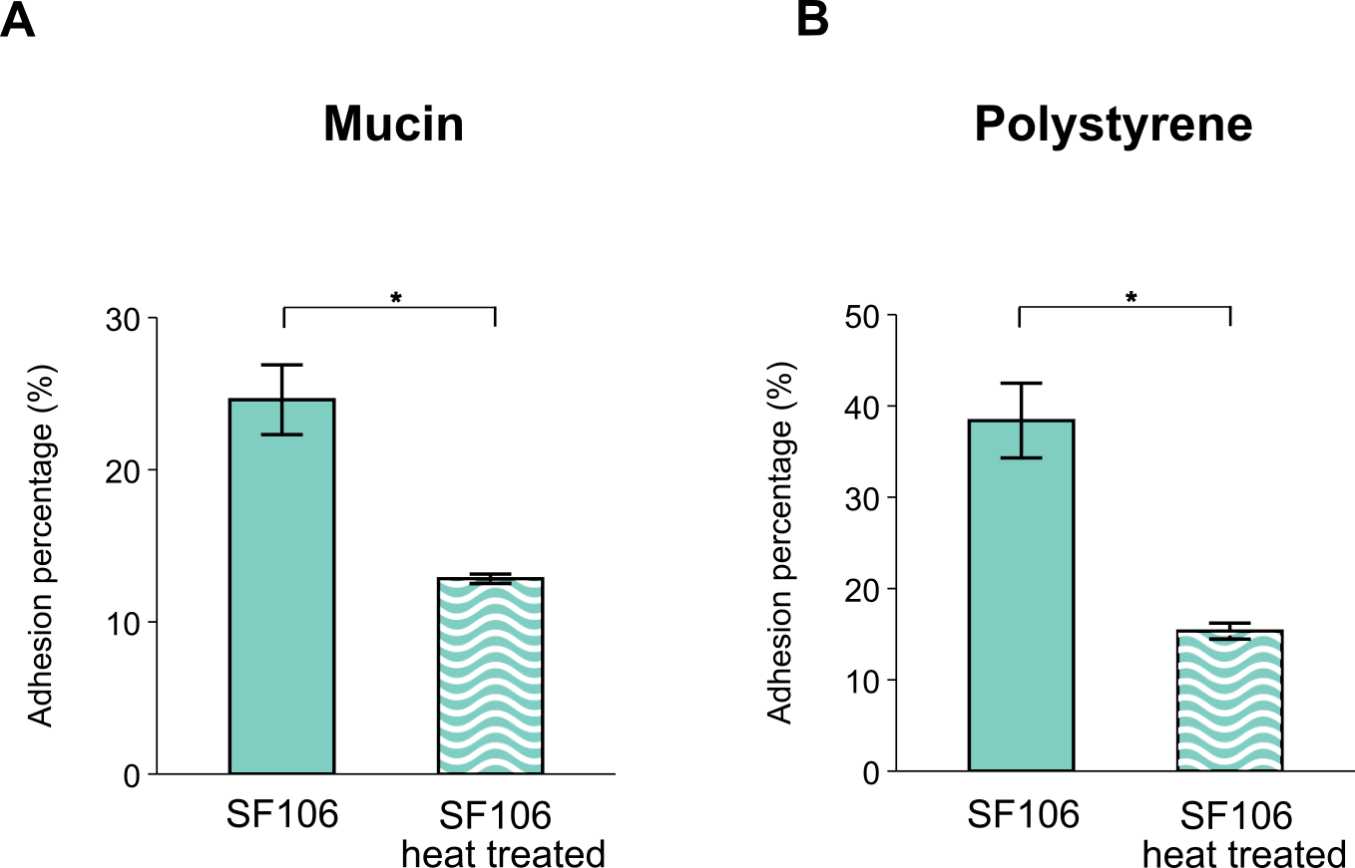
Adhesion of spores of strain SF106 produced with or without a heat-treatment aimed at killing vegetative cells (not committed to sporulate) in the heterogeneous culture to human mucin (**A**) and to polystyrene (**B**). The heat-treatment (80°C for 20 min) was performed after 20 h of growth. Growth continued after the heat-treatment for additional 28 h. The data represent the mean of three independent experiments, and the error bars are the standard error of the mean (*P < 0.05).

To test the hypothesis that the EPS, once released by non-sporulating cells, attached to the spore surface, two experimental approaches were followed. A first strategy consisted in checking whether the elimination/reduction of vegetative cells, not committed to sporulate, present in the culture affected the adhesion properties of the produced spores. To this aim, SF106 cells grown in DSM for 20 h were observed under the light microscope and were shown to still contain approximately 50% of vegetative cells. Half of the DSM culture was heat-treated (80°C for 20 min) to kill the vegetative cells and then incubated for additional 28 h (up to 40 h after the onset of sporulation) in the same medium. The other half of the culture was not heat-treated and grown for a total of 48 h. Spores were then purified from the two parallel cultures and used to evaluate their adhesion efficiencies. As shown in Fig. 9, spores produced by the heat-treated culture were significantly less adhesive than an identical number of spores produced in the untreated culture to both biotic and abiotic surfaces.

**Fig 10:**
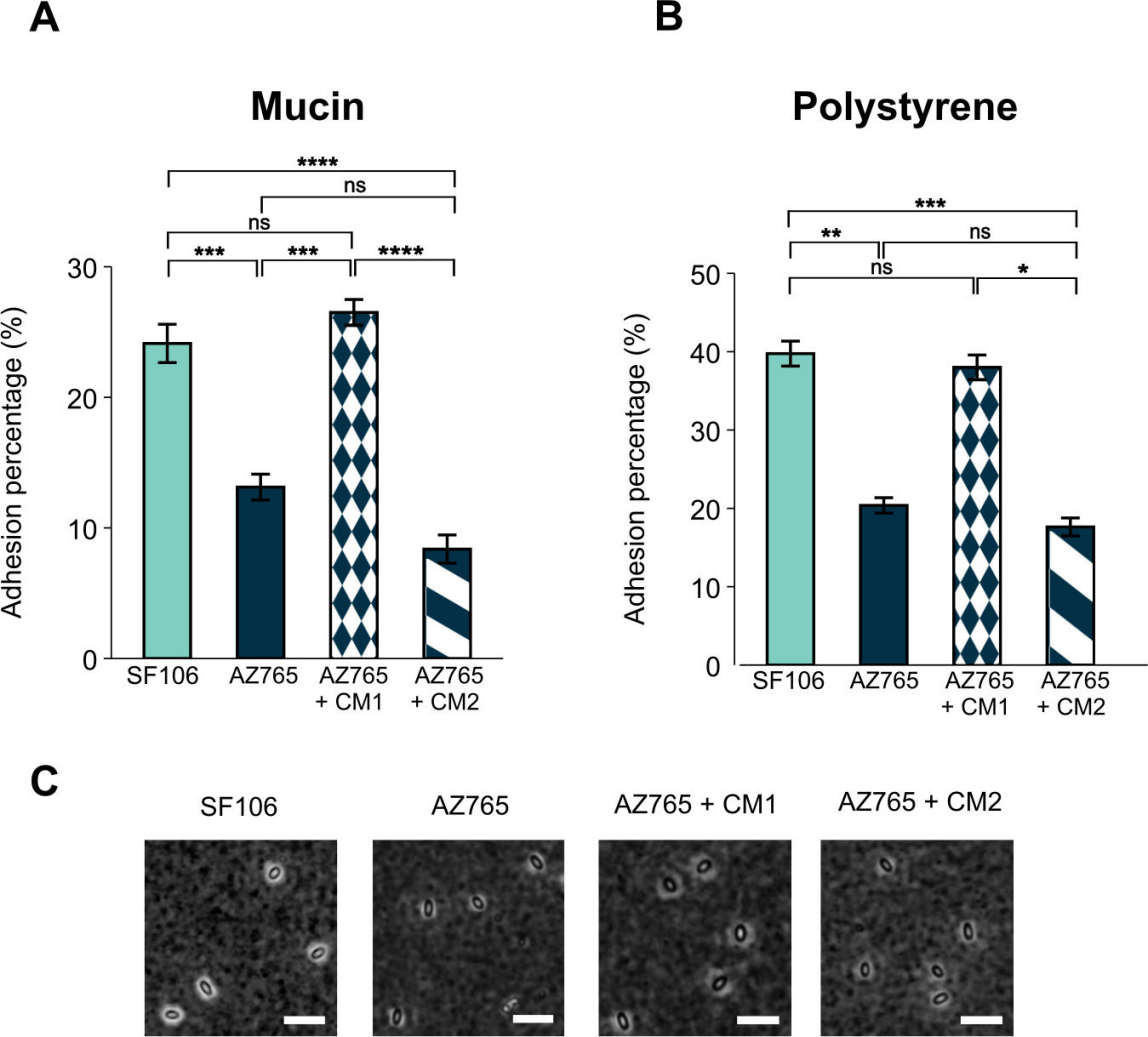
Adhesion of spores to mucin (**A**) and polystyrene (**B**) of strains SF106 and AZ765 (*ydaJKLMN*) supplemented or not with cell-free conditional medium (CM) produced by strains AZ772 (*spo0A::neo*) (CM1) and AZ792 (*ydaJKLMN::cm spo0A::neo*) (CM2). The data represent the mean of three independent experiments, and the error bars are the standard error of the mean (ns: differences not statistically significant; *P < 0.05; **P < 0.005; ***P < 0.001; ****P < 0.0001). (**C**) Negative staining of *B. subtilis* spores with India Ink (Size bar = 4 µm).

A second approach consisted in checking whether the cell-free supernatant of a YdaJKLMN-producing cell culture (SF106) in DSM was able to increase the efficiency of adhesion of spores of a strain lacking *ydaJKLMN* (AZ765). To this aim, a strain unable to produce spores was used to avoid that the produced polysaccharides were immediately bound to spores produced in the same culture. Therefore, the cell-free conditioned medium (CM) of strains AZ772 (*spo0A::neo*) and AZ792 (*ydaJKLMN::cm spo0A::neo*) was collected after 40 h of growth in DSM and added to spores of a low adhesive strain (AZ765). As shown in Fig. 10A and B, only the CM of a YdaJKLMN-expressing strain (CM1 in the figure) was able to increase the adhesion of AZ765 spores to levels similar to those of strain SF106. The CM of a YdaJKLMN lacking strain (CM2 in Fig. 10A and B) failed to increase the adhesion of AZ765 spores to biotic and abiotic surfaces indicating that the observed effects were due to the presence of a functional *ydaJKLMN* operon. Further support to the hypothesis came from the observation that AZ765 spores were recognized by the lectin GSL-1 only when mixed with the CM of a YdaJKLMN-expressing strain (CM1) (Table 3).

**TABLE 3:** Lectin-binding analysis^*a*^

	SF106	AZ765	AZ765 + CM1	AZ765 + CM2
GSL-1	+	−	+	−

^*a*^ *Griffonia simplicifolia* lectin I (GSL-I): α-D-galactosyl and α-N-acetyl-D-galactosaminyl (GalNAc) residues. Presence (+) or absence (−) of fluorescence signals after 400 ms of exposure time.

Finally, phase contrast microscopy analysis after negative staining with indian inkshowed a larger and brighter polysaccharide halo around SF106 than that observed with AZ765 spores (Fig. 10). The addition of CM1 but not CM2 increased the size of the halo around AZ765 spores (Fig. 10C).

## DISCUSSION

The main conclusion of this work is that *B. subtilis* strains containing the *ydaJKLMN* operon form spores more adhesive to biotic and abiotic surfaces than spores produced by strains that do not contain that operon.

Products of the *ydaJKLMN* operon catalyze the synthesis of an exopolysaccharide (EPS) (32). The promoter-proximal gene of the operon codes for a glycosyltransferase localized on the outer side of the cell membrane and probably not essential for EPS production but involved in its modification (32, 44). The second gene, *ydaK*, encodes a membrane-bound c-di-GMP binding protein (45), essential for the regulation of EPS biosynthesis (32, 44). The other three genes, *ydaLMN*, are also essential for EPS production and are likely responsible of the catalytic activities for EPS synthesis (32, 44). Interestingly, the YdaJKLMN proteins form a subcellular cluster at the cell poles, with YdaJ and YdaM localized on the outer side and YdaKMN on the cytoplasmic side of the membrane (32, 44). Based on the observation that the operon is controlled by the stress-specific, alternative sigma factor SigB (32, 35, 44), the YdaJKLMN-dependent EPS has been so far suggested to protect vegetative cells when they are under stress conditions. Results present here link the YdaJKLMN-dependent EPS with spore adhesion, therefore highlighting an additional role for the EPS produced by the expression of the *ydaJKLMN* operon.

Results of this work confirm that *ydaJKLMN* is under SigB control and indicate that no sporulation-specific transcription factors are involved in its expression. Indeed, *ydaJKLMN* expression is higher in a biofilm-inducing medium (MSgg) than in a sporulation-inducing medium (DSM), there are no internal promoters contributing to the expression of the operon (same β-galactosidase activity of the *ydaJ::lacZ* and *ydaN::lacZ* fusions), and the lack of sporulation-specific factors (in a *spo0A* null background) does not affect *ydaJKLMN* expression.

The results of this study raise a paradox: the products of an operon that is not expressed by sporulating cells have an effect on the produced spores. The proposed explanation of such apparent paradox is based on the well-established idea that in a sporulation-inducing medium there is a heterogeneous population of cells, in which sporulating and not-sporulating cells co-exist (41, 42). Only the not-sporulating cells produce a EPS that, once released, binds to spores produced by neighbor cells modifying their phenotype (adhesion properties). Single-cell analysis confirmed that only cells not committed to sporulate expressed the *ydaJKLMN* operon and two lines of evidence support such a model: (i) when not-sporulating cells are killed by a heat-treatment the adhesion efficiency of the produced spore is similar to that of spores produced by a strain lacking the *ydaJKLMN* operon; (ii) when the cell-free exhausted medium of *ydaJKLMN*-containing strain is added to spores produced by a *ydaJKLMN*-lacking strain spores are more adhesive and are recognized by the GSL-1 lectin, specifically able to recognize the *ydaJKLMN-*dependend EPS. Based on all this, the effects of *ydaJKLMN* on spore adhesion efficiency observed in this study could be incidental and due to EPS secretion by co-existing non-sporulating cells. However, such an heterogeneous situation with cells in a different physiological condition (and expressing different programs of gene expression) co-existing in the same *in vitro* environment most likely resembles a natural condition more closely than a homogeneous culture of metabolically identical cells. The observed phenomenon could then be a common behavior of microbial populations in a natural environment, with the products of the metabolic activities of some cells used by other cells to expand their own biological functions.

## MATERIALS AND METHODS

### Bacterial strains and growth conditions

All bacterial strains used in this study are listed in Table S1. *Escherichia coli* DH5α was employed for plasmid amplification, nucleotide sequencing, and subcloning experiments (46). Transformation of *E. coli* competent cells was performed by the CaCL_2_-mediated method (46), whereas *B. subtilis* strains were transformed using the previously described two-step procedure (47). DNA ligation, plasmid isolation, and restriction enzyme digestion were carried out according to standard protocols. Chromosomal DNA from *B. subtilis* was purified following established methods (46).

*E. coli* and *B. subtilis* strains were routinely grown in Luria-Bertani medium (8 g/L NaCl, 10 g/L tryptone, 5 g/L yeast extract; Difco) at 37°C. For biofilm-inducing conditions, *B. subtilis* cultures were grown in MSgg medium (5 mM potassium phosphate [pH 7], 100 mM Mops [pH 7], 2 mM MgCl_2_, 700 μM CaCl_2_, 50 μM MnCl_2_, 50 μM FeCL_3_, 1 μM ZnCl_2_, 2 μM thiamine, 0.5% glycerol, 0.5% glutamate, and 50 μg mL^−1^ each of tryptophan, phenylalanine, and threonine), adapted from reference 48. When required, antibiotics were added at the following concentrations: 5 µg mL^−1^ chloramphenicol, 10 µg mL^−1^ kanamycin, and 5 µg mL^−1^ neomycin.

### Strain construction

Gene deletions and transcriptional fusions were generated using standard PCR-based cloning strategies, followed by integration of recombinant plasmids or chromosomal DNA into competent cells of *B. subtilis* via natural transformation (46). Unless otherwise stated, PCR amplifications were performed using Taq DNA polymerase (Thermo Fisher Scientific) in a Biometra TPersonal Thermocycler (Analytik Jena) for 30 cycles (30 s at 95°C, 30 s at annealing temperature, 45 s at 72°C). PCR products were first cloned into the pGEM-T Easy vector (Promega), sequenced, and subsequently subcloned into integrative plasmids carrying antibiotic-resistance cassettes. Recombinant plasmids or chromosomal DNA were then used to transform competent cells of *B. subtilis*, and transformants were selected on LB agar supplemented with the appropriate antibiotic.

Deletion of *ydaJ, sigB,* or *spo0A* genes was achieved by amplifying internal fragments of each open reading frame by using gene-specific primers carrying restriction sites. For *ydaJ*, a 698-bp internal fragment (positions 95–793 of the SF106 *ydaJ* gene) was amplified using primers ydaKO_For and ydaKO_Rev (Table S2). After cloning into pGEM-T Easy (Promega), the fragment was excised with *Bam*HI and *Eco*RI and ligated into vector pER19 carrying a gene conferring chloramphenicol-resistance, generating plasmid pSO16. Transformation of competent *B. subtilis* SF106 cells with pSO16 yielded the *ΔydaJ* strain AZ765.

An internal region of the *sigB* gene (604 bp; positions 121–724 of the *B. subtilis* SF106 sequence) was amplified using primers SigmaBD_F_KpnI and SigmaBD_R_BamHI (Table S2). The fragment was digested with *Kpn*I and *Bam*HI and cloned into pNEO, a neomycin-resistant integrative vector, generating plasmid pMDS6. Transformation of *B. subtilis* SF106 with pMDS6 produced the *ΔsigB* strain AZ771.

Similarly, a 594-bp internal fragment of *spo0A* gene (positions 87–680) was amplified using primers Spo0AD_F_KpnI and Spo0AD_Rev_BamHI (Table S2), digested with *Kpn*I and *Bam*HI, and cloned into pNEO, yielding plasmid pMDS7. Transformation of *B. subtilis* SF106 with pMDS7 generated the *Δspo0A* strain AZ772.

### Construction of transcriptional fusions

Transcriptional *lacZ* fusions were generated using the integrative reporter vector pJM783*,* which carries a promoter-less *lacZ* gene and a chloramphenicol-resistance cassette (49).

For the *ydaJ::lacZ* fusion, a 698-bp internal fragment of the *ydaJ* region (positions 201–942) was amplified with primers ydaKO_For and ydaKO_Rev (Table S2), cloned into pGEM-T Easy (Promega)*,* excised with *Eco*RI, and ligated into pJM783, generating plasmid pMDS1. Transformation of competent *B. subtilis* SF106 cells produced strain AZ769.

For the *ydaN::lacZ* fusion, a 742-bp internal region of *ydaN* (positions 95–793) amplified with primers ydaN_for_EcoRI and ydaN_Rev_BamHI (Table S2) was cloned into pGEM-T Easy (Promega)*,* yielding plasmid pMDS2, and then subcloned into pJM783 using *Eco*RI and *Bam*HI, generating plasmid pMDS3. Transformation of SF106 yielded strain AZ770.

To obtain recombinant strains carrying the gene fusions but lacking sporulation regulators, chromosomal DNA from strains AZ771 (*ΔsigB::neo*) and AZ772 (*Δspo0A::neo*) was used to transform competent cells of strains AZ769 (*ydaJ::lacZ*) and AZ770 (*ydaN::lacZ*), generating strains AZ773 (*ydaJ::lacZ ΔsigB*), AZ774 (*ydaN::lacZ ΔsigB*), AZ775 (*ydaJ::lacZ Δspo0A*), AZ776 (*ydaN::lacZ Δspo0A*).

### Construction of RFP fusion

A *ydaJ::rfp* fusion was constructed by cloning a 678 bp DNA fragment encoding the monomeric red fluorescent protein (mRFP) (43) into the plasmid pSO16. This plasmid already carried a 698 bp internal fragment of the *ydaJ* gene (positions 95–793 of the SF106 *ydaJ* gene) along with a chloramphenicol-resistance cassette. The *rfp* sequence was cloned in frame with the *ydaJ* start codon (ATG) by inserting a PCR-amplified fragment with primers *ydaJ::rfp_F* and *ydaJ::rfp_R* (Table S2) into the linearized pSO16 vector. The cloning was performed using the In-Fusion Snap Assembly Cloning Kit (Takara Bio USA, Inc.), resulting in the derived plasmid pMDS10. Competent cells of strain SF106 were transformed with plasmid pMDS10, obtaining strain AZ804 (*ydaJ::rfp*).

### Construction PY79-derivative strains carrying the *ydaJKLMN* operon

Competent cells of strain PY79 were transformed with chromosomal DNA from strain BKK04206 (BGSC), which carries a kanamycin cassette inserted downstream of *topB* (topB::kan), yielding strain AZ766. Subsequently, AZ766 cells were transformed with plasmid pSO16 (*ΔydaJ* construct), generating strain AZ767, a PY79 derivative carrying both *topB::kan* and *ΔydaJ*. Competent cells of strain AZ772 (*spo0A::neo*) were transformed with plasmid pSO16, yielding strain AZ792 (*spo0A::neo ydaJ*).

### Spore purification

Sporulation of *B. subtilis* strains was induced by the exhaustion method (50) in Difco Sporulation Medium (DSM; 8 g L^−1^ nutrient broth, 1 g L^−1^ KCl, 1 mM MgSO_4_, 1 mM Ca(NO_3_)_2_, 10 µM MnCl_2_, 1 µM FeSO_4_; Sigma-Aldrich, Germany) and incubated at 37°C with vigorous shaking. After ~40 h, sporulation efficiency was evaluated microscopically. Spores were harvested by centrifugation, washed four times with distilled water, and purified by lysozyme treatment (47). Purity was verified microscopically, and preparations containing <5% sporangia were considered pure.

### Preparation of cell-free conditioned media

To obtain cell-free supernatants enriched (or not enriched) in YdaJKLMN-dependent exopolysaccharides, two *B. subtilis* strains defective in sporulation were used: AZ772 (*spo0A::neo*), expressing an intact *ydaJKLMN* operon, and AZ792 (*ydaJKLMN::cm spo0A::neo*), unable to synthesize the exopolysaccharide.

Strains were inoculated in DSM medium and incubated at 37°C with shaking (120 rpm) for 40 h. Cultures were then centrifuged at 3,000 rpm for 10 min at room temperature, and the resulting supernatants were carefully transferred to sterile tubes. To remove any residual cells, supernatants were filtered through 0.22 µm PES membrane filters (EuroClone), generating the corresponding conditioned media (CM1 from AZ772 and CM2 from AZ792). Conditioned media were used immediately or stored at 4°C for a maximum of 24 h.

### Incubation of spores with conditioned media

Aliquots containing 5 × 10^7^ spores were pelleted by centrifugation (10,000 rpm, 5 min) and resuspended in 1 mL of either CM1 or CM2. Suspensions were incubated at 25°C for 1 h under mild agitation (100 rpm). After incubation, spores were collected by centrifugation, washed twice with sterile PBS to remove unbound polysaccharides, and resuspended in PBS to the original volume.

### Mucin and polystyrene adhesion assays

Adhesion assays were performed as described previously (51) with minor modifications. Ninety-six-well microtiter plates were coated overnight at 4°C with 3 mg mL^−1^ mucin from porcine stomach (Sigma-Aldrich, USA). Wells were incubated with 40 µL of spore suspensions (5 × 10^6^ spores per well) for 3 h at 37°C. Unbound spores were removed by three washes with 100 µL PBS, and bound spores were released by incubation with 200 µL of 0.06% Triton X-100 for 30 min at 37°C. Serial dilutions were plated on LB agar and incubated 24 h at 37°C; colony-forming units (CFU mL^−1^) were then determined. Total spore counts were determined from unwashed mucin-coated wells.

For the polystyrene adhesion assay, the same procedure was followed except that spore suspensions were directly added to uncoated 96-well plates. All assays were performed in at least triplicate.

Adhesion percentages from three independent experiments were compared across strains using a Kruskal-Wallis nonparametric test, followed by Dunn’s multiple comparisons test with adjusted *P*-values. Statistical significance was defined as *P* < 0.05.

### Lectin labeling of spores

Purified spores were resuspended in PBS1x and TRITC-labeled lectins (Vector Laboratories) were applied at a concentration of 20 μg/mL. Spores were incubated with lectins overnight at 4°C, washed twice with PBS, and subsequently deposited on a glass slide for phase contrast and fluorescence microscopy. Spores presenting red fluorescence were considered positive for the specific lectin-sugar binding.

### RFP adsorption

Adsorption reactions were carried out as previously described (38). Two micrograms of purified mRFP was added to a suspension of 1 × 10^9^ spores in 50 mM sodium citrate buffer (pH 4.5) in a final volume of 200 µL. After 1 h of incubation at 25°C, the mixtures were centrifuged (13,000 rpm, 10 min) to separate spore-bound (pellet) from unbound (supernatant) protein fractions. The pellet fraction was analyzed by fluorescence microscopy.

### Fluorescence microscopy

To visualize mRFP display on the spore surface, 6 µL of spore suspension was spotted onto glass slides coated with 0.1% poly-L-lysine (Sigma-Aldrich) and covered with a coverslip. Samples were examined under an Olympus BX51 microscope equipped with a 100 × UPlanF1 objective and TRITC filters (U-MWIG2). Red fluorescence was captured using 500 ms exposure time. Images were processed with analySIS software (SIS GmbH) and analyzed using ImageJ (https://imagej.net/ij/) to calculate the corrected total cell fluorescence (CTCF). Differences among strains were assessed using Kruskal-Wallis test followed by Dunn’s post-hoc multiple comparisons test. Statistical significance was defined as *P* < 0.05.

### India ink staining

Spores were resuspended in dH_2_O. Five microliters was mixed with an equal amount of India ink (Winsor & Newton Ink) and observed by phase-contrast microscopy as described previously (52).

### β-Galactosidase assays

β-Galactosidase activity was assayed as described (53). Cells were grown in LB medium at 37°C with shaking, and 10 mL of the culture was used to inoculate MSgg or DSM medium, followed by incubation at 37°C. Samples (1 mL) were collected at the indicated time points, centrifuged (5,000 rpm, 10 min), and resuspended in 1 mL Z buffer (40 mM NaH_2_PO_4_, 60 mM Na_2_HPO_4_, 1 mM MgSO_4_, 10 mM KCl, 38 mM β-mercaptoethanol) containing 0.01% toluene. After 30 min incubation at 30°C, reactions were initiated by adding 200 µL of 4 mg mL^−1^ ONPG (2-nitrophenyl-β-D-galactopyranoside) and terminated by adding 500 µL of 1 M Na_2_CO_3_. Following centrifugation (5,000 rpm, 10 min), absorbance at 420 nm was recorded. Specific β-galactosidase activity was calculated as (*A*_420_/ [reaction time × OD_600_]) ×1.000. All assays were performed in at least triplicate. To compare transcriptional activation dynamics across genetic backgrounds, β-galactosidase values (Miller Units) were modeled as a function of time using linear regression. For each strain, the regression slope was taken as a quantitative descriptor of promoter activity over time. A linear model including strain, time, and a time × strain interaction term was fitted to test whether strains differed in their temporal trends. Post-hoc pairwise comparisons of slopes were obtained using estimated marginal trends (emtrends) from the emmeans package (R v.4.3), and *P*-values were adjusted for multiple testing using the Tukey HSD method. Statistical significance was defined as *P* < 0.05.

### Pangenome analysis

A comparative genomic analysis was performed with genomes of *B. subtilis* SF106 and the type strains belonging to the same species 168, NBCI 3610, PY79, using the Anvi’o Pangenome workflow (version 8) (54). Briefly, a database was generated using Prodigal (version 2.6.3) (55) to identify open reading frames in the contigs. Then, to enrich the contig database with additional details, the NCBI Clusters of Orthologous Groups (COG) (56) database were used to assign gene functions, and the KEGG KOfam database was used to annotate the genes. Finally, the tools Spine and AGEnt were employed to determine the core and accessory genes.

## Data Availability

The data that support the findings of this study are available in the article and its supplemental material.

## SUPPLEMENTAL MATERIAL

Fig. S1 aem.00086-26-s0001.pdf Construction of a null mutation.

Supplemental tables aem.00086-26-s0002.docx Tables S1 to S4.

ASM does not own the copyrights to Supplemental Material that may be linked to, or accessed through, an article. The authors have granted ASM a non-exclusive, world-wide license to publish the Supplemental Material files. Please contact the corresponding author directly for reuse.
