# Supplementary material for: Expression of the ydaJKLMN operon increases the adhesion of Bacillus subtilis spores to biotic and abiotic surfaces
Source: Appl Environ Microbiol. 2026 May 27;92(6):e00086-26. doi: 10.1128/aem.00086-26 (PMC13274460; doi:10.1128/aem.00086-26)
Supplement: Fig. S1 — Construction of a null mutation. [file aem.00086-26-s0001.pdf]

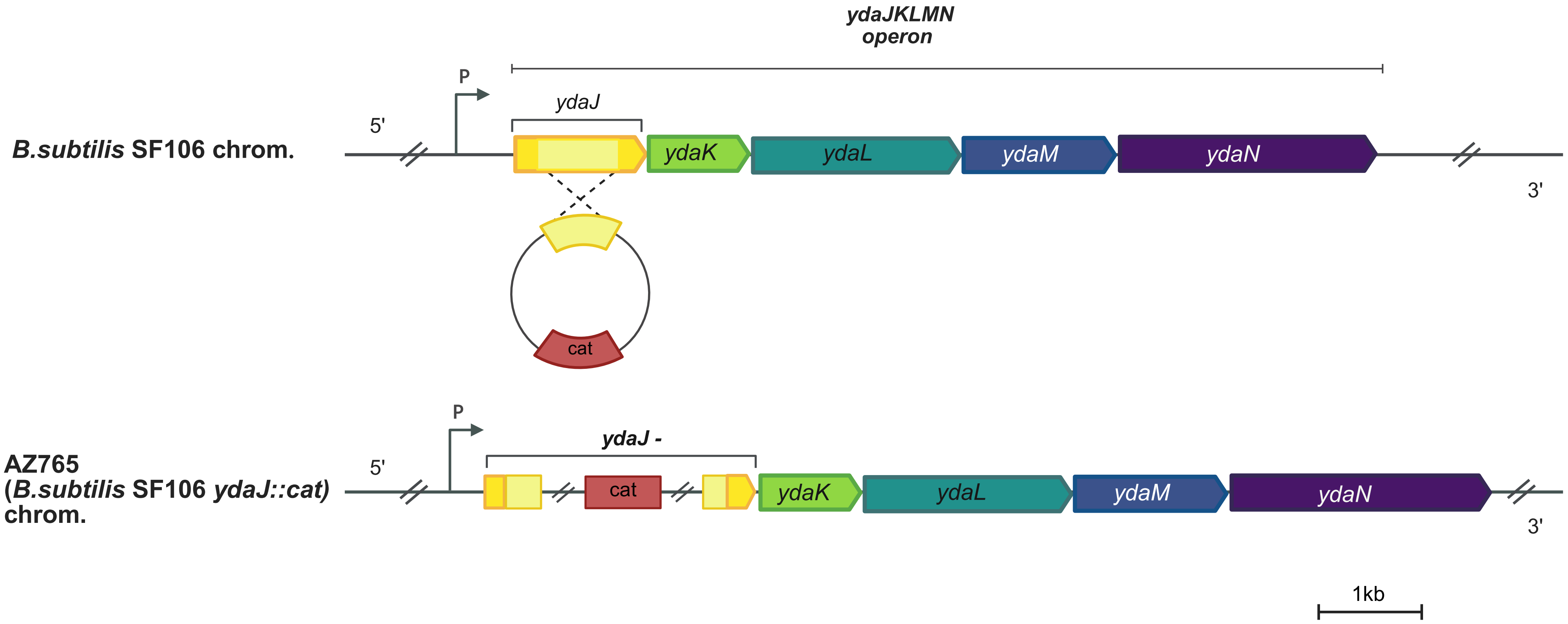

Fig. s1 Construction of a null mutation in the *ydaJKLMN* operon.
Created in BioRender. De stefano, M. (2026) https://BioRender.com/5po4d7c
