# Supplementary material for: Expression of the ydaJKLMN operon increases the adhesion of Bacillus subtilis spores to biotic and abiotic surfaces
Source: Appl Environ Microbiol. 2026 May 27;92(6):e00086-26. doi: 10.1128/aem.00086-26 (PMC13274460; doi:10.1128/aem.00086-26)
Supplement: Supplemental tables — Tables S1 to S4. [file aem.00086-26-s0002.docx]

**Table S1.** List of strains used in this study

| **Strain (background)** | **Relevant genotype** | **References** |
| --- | --- | --- |
| PY79 | Wild type | [Youngman et al, 1984] |
| 168 | Wild type | [Hederstedt et al, 1982] |
| NCIB 3610 | Wild type | [Dean et al, 1978] |
| SF106 | Wild type | [Fakhry et al, 2008] |
| BKK04260 | *topB::kan* | *Bacillus* Genetic Stock Centre |
| AZ765 (SF106) | *ydaJ::cm* | This study |
| AZ766 (PY79) | *topB::kan* (17 kb region) | This study |
| AZ768 (PY79) | *topB::kan* (17 kb region) *ydaJ::cm* | This study |
| AZ769 (SF106) | *ydaJ::lacZ* | This study |
| AZ770 (SF106) | *ydaN::lacZ* | This study |
| AZ771 (168) | *sigB::neo* | This study |
| AZ772 (168) | *spo0A::neo* | This study |
| AZ773 (SF106) | *ydaJ::lacZ; sigB::neo* | This study |
| AZ774 (SF106) | *ydaN::lacZ; sigB::neo* | This study |
| AZ775 (SF106) | *ydaJ::lacZ;*  *spo0A::neo* | This study |
| AZ776 (SF106) | *ydaN::lacZ; spo0A::neo* | This study |
| AZ792 (168) | *ydaJ::cm; spo0A::neo* | This study |
| AZ804 (SF106) | *ydaJ::rfp* | This study |

**Table S2.** Oligonucleotides used in this study

| **Primer** | | **Sequence 5' - 3' ^(a)^** | **Restriction site** | **Position of**  **annealing** |
| --- | --- | --- | --- | --- |
| *yda*KO_For | ggatccGGCGACAGAAGTGAATG | BamHI | 95/113 (*ydaJ*) |  |
| *yda*KO_Rev | gaattcGCTTGATCCAGTCCGC | HindIII | 777/793 (*ydaJ*) |  |
| *SigmaB*D_F_KpnI | ggtaccGCAAGATGAACAAGCGCAG | KpnI | 121/138 (*sigB*) |  |
| *SigmaB*D_R_BamHI | ggatccCTTCACAGCTTTGCGTTGC | BamHI | 708/724 (*sigB*) |  |
| *Spo0A*D_F_KpnI | ggtaccGGAAGTGATCGGCGTTGC | KpnI | 87/104 (*spo0A*) |  |
| *Spo0A*D_Rev_BamHI | ggatccACCTCAGCTTATCCGCAACC | BamHI | 664/680 (*spo0A*) |  |
| *ydaN*_For_EcoRI | gaattcGGCAGGCGCCTCTTCG | EcoRI | 201/216 (*ydaN*) |  |
| *ydaN*_Rev_BamHI | ggatccGCCGCTGAGCTGGCC | BamHI | 928/942 (*ydaN*) |  |
| *ydaJ::rfp_F* | TGGATCAGCGAATTATGGCCTCCTCCGAGGAC | EcoRI | 1/18 (*rfp*) |  |
| *ydaJ::rfp_R* | GACGGCCAGTGAATTTTAGGCGCCGGTGGAGTG | EcoRI | 662/678 (*rfp*) |  |

(a)Capital and lowercase letters indicate bases of DNA and of an unpaired tail carrying a restriction site, respectively.

**Table S3.** Pairwise comparisons of slopes for β-galactosidase expression in MSgg.

| **Comparison in MSgg*** | **Difference in slope** | **Significance** |
| --- | --- | --- |
| *ydaJ vs ydaJ sigB⁻* | 0.4750 | * |
| *ydaJ vs ydaJ spo0A⁻* | 0.0025 | ns |
| *ydaJ vs ydaN* | 0.0150 | ns |
| *ydaJ vs ydaN sigB⁻* | 0.4800 | * |
| *ydaJ vs ydaN spo0A⁻* | –0.0075 | ns |
| *ydaJ sigB⁻ vs ydaJ spo0A⁻* | –0.4725 | * |
| *ydaJ sigB⁻ vs ydaN* | –0.4600 | * |
| *ydaJ sigB⁻ vs ydaN sigB⁻* | 0.0050 | ns |
| *ydaJ sigB⁻ vs ydaN spo0A⁻* | –0.4825 | * |
| *ydaJ spo0A⁻ vs ydaN* | 0.0125 | ns |
| *ydaJ spo0A⁻ vs ydaN sigB⁻* | 0.4775 | * |
| *ydaJ spo0A⁻ vs ydaN spo0A⁻* | –0.0100 | ns |
| *ydaN vs ydaN sigB⁻* | 0.4650 | * |
| *ydaN vs ydaN spo0A⁻* | –0.0225 | ns |
| *ydaN sigB⁻ vs ydaN spo0A⁻* | –0.4875 | * |

***** Strain names are abbreviated: “ydaJ” and “ydaN” indicate the transcriptional fusions *ydaJ::lacZ* and *ydaN::lacZ*, respectively; “sigB⁻” = *sigB::neo*; “spo0A⁻” = *spo0A::neo*. Combined labels (e.g., “ydaJ sigB⁻”) refer to the corresponding double mutants (*ydaJ::lacZ sigB::neo*, etc.).

**Table S4.** Pairwise comparisons of slopes for β-galactosidase expression in DSM.

| **Comparison in DSM *** | **Difference in slope** | **Significance** |
| --- | --- | --- |
| *ydaJ vs ydaJ sigB⁻* | 0.1125 | trend (ns) |
| *ydaJ vs ydaJ spo0A⁻* | –0.0875 | ns |
| *ydaJ vs ydaN* | –0.0500 | ns |
| *ydaJ vs ydaN sigB⁻* | 0.1275 | * |
| *ydaJ vs ydaN spo0A⁻* | –0.0700 | ns |
| *ydaJ sigB⁻ vs ydaJ spo0A⁻* | –0.2000 | ** |
| *ydaJ sigB⁻ vs ydaN* | –0.1625 | ** |
| *ydaJ sigB⁻ vs ydaN sigB⁻* | 0.0150 | ns |
| *ydaJ sigB⁻ vs ydaN spo0A⁻* | –0.1825 | * |
| *ydaJ spo0A⁻ vs ydaN* | 0.0375 | ns |
| *ydaJ spo0A⁻ vs ydaN sigB⁻* | 0.2150 | ** |
| *ydaJ spo0A⁻ vs ydaN spo0A⁻* | 0.0175 | ns |
| *ydaN vs ydaN sigB⁻* | 0.1775 | * |
| *ydaN vs ydaN spo0A⁻* | –0.0200 | ns |
| *ydaN sigB⁻ vs ydaN spo0A⁻* | –0.1975 | ** |

***** Strain names are abbreviated: “ydaJ” and “ydaN” indicate the transcriptional fusions *ydaJ::lacZ* and *ydaN::lacZ*, respectively; “sigB⁻” = *sigB::neo*; “spo0A⁻” = *spo0A::neo*. Combined labels (e.g., “ydaJ sigB⁻”) refer to the corresponding double mutants (*ydaJ::lacZ sigB::neo*, etc.).
